# Update on human papillomavirus – Part II: complementary diagnosis, treatment and prophylaxis^☆^^☆☆^

**DOI:** 10.1016/j.abd.2020.11.005

**Published:** 2021-02-16

**Authors:** Marcelo Grossi Araújo, Geraldo Magela Magalhães, Lucas Campos Garcia, Érica Cristina Vieira, Maria de Lourdes Ribeiro de Carvalho-Leite, Antônio Carlos Martins Guedes

**Affiliations:** a Department of Internal Medicine, Faculty of Medicine, Universidade Federal de Minas Gerais, Belo Horizonte, MG, Brazil; b Dermatology Service, Hospital das Clínicas, Universidade Federal de Minas Gerais, Belo Horizonte, MG, Brazil; c Secretaria de Estado de Saúde de Minas Gerais, Belo Horizonte, MG, Brazil

**Keywords:** Papillomaviridae, Papillomavirus infections, Papillomavirus infections/diagnosis, Papillomavirus infections/drug therapy, Papillomavirus infections/pathology, Papillomavirus infections/prevention & control

## Abstract

In this nonsystematic review, the complementary diagnosis, treatment, prevention, and control of *human papillomavirus* are discussed. The histopathology is addressed regarding its indications, main findings and limitations, as a complementary diagnostic method largely used by dermatologists. Electron microscopy is briefly reviewed, along with its contribution to the accumulated knowledge on HPV, as well as the relevance of research in using this technology for future advances in diagnosis and treatment. Molecular information about the virus is continuously increasing, and the practical applications of HPV serology, molecular identification and genotyping are discussed. Vaccines are a valuable tool in primary HPV infection prevention and are now available in many countries; their composition, indications, and adverse effects are revisited. Local and systemic treatment options are reviewed and off-label prescriptions are discussed. Finally, health education focusing on HPV infection as a sexually transmitted infection of worldwide relevance and the many barriers to improve primary and secondary prevention are addressed.

## Introduction

In a previous article, aspects of epidemiology, pathogenesis and the main clinical manifestations of the Human Papilloma Virus (HPV) infection were discussed.

This non-systematic review article addresses the topics of complementary diagnosis, treatment and preventive measures.

## Complementary diagnosis

### Histopathology and electron microscopy

The histopathological analysis of lesions caused by HPV should be considered whenever there is doubt regarding the differential diagnosis and also when malignancy is suspected, both on the skin and on the mucosal membranes. Oncotic colpocytology remains recommended as a periodic examination for Cervical Cancer (CC) screening.^1^ There is evidence that cytopathology for the follow-up of patients at high risk for the development of anal cancer is cost-effective; however its routine use is still restricted.^2^

HPVs are known to infect epithelial cells and depend on epithelial cells differentiation to complete their life cycle.^3^ Moreover, the expression of the virus gene products is regulated as the infected basal cell migrates to the epithelial surface. The expression of proteins E6 and E7 at the bottom of the epithelial layers, directs cells to the S phase of the cell cycle, which creates a favorable environment for viral genome replication and cell proliferation. Consequently, there would be a stimulus for epithelial hyperplasia that would result, from the histopathological point of view, in acanthosis and papillomatosis.^4^

### Histopathology of skin lesions

#### Verruca vulgaris

**Figure 1:**
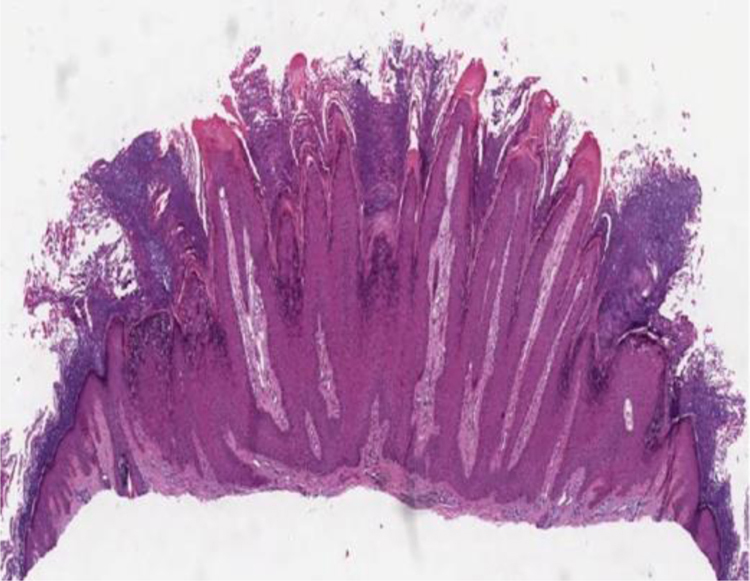
Verruca vulgaris or common wart showing hyperkeratosis, papillomatosis, acanthosis and rete ridges with their axis inclined toward the center of the lesion (Hematoxylin & eosin, 40×).

The histopathological characteristics of verruca vulgaris or common warts are evident in the epidermis, where the presence of acanthosis, papillomatosis, marked hyperkeratosis, and parakeratosis columns can be observed on the top of the epithelial cones, with the absence of the granular layer that is accentuated in the valleys between adjacent cones.^5^ In the areas of parakeratosis, small accumulations of blood or serosity can be observed.^6^ The elongated epithelial cones at the border of the lesion exhibit an internal curvature at the base toward the center of the lesion, referred to as arborization (Fig. 1).

The three histopathological features that are used to differentiate the common wart from other papillomas include the presence of foci of vacuolated cells, known as koilocytes, vertical columns of parakeratosis and dense foci of keratohyalin granules. These characteristic epidermal alterations are best seen in young warts.

Koilocytes (from the Greek *koilos*/cavity), which are characteristic of HPV infection, are observed in the upper stratum of Malpighian layer and in the stratum granulosum; they are characterized by small, round and dense basophilic nuclei, surrounded by a clear halo with pale cytoplasm. Sometimes, these lesions can resemble seborrheic keratoses due to the loss of their characteristic shape. Dermal alterations are usually minimal in the common wart. Approximately 8% are inflamed and show a lichenoid infiltrate. Filiform warts show much more evident papillomatosis with marked hyperkeratosis, dilated capillaries in the dermis and, sometimes, small hemorrhagic foci.^5^

#### Palmoplantar warts

In the histopathological analysis, palmoplantar warts have elements in common with the common wart; however, they have an endophytic growth with a large part of the lesion penetrating the dermis, below the epidermis. There are differences in the appearance of the eosinophilic keratohyalin granules seen in the spinous layer cells, depending on the type of HPV. HPV Type 1 infections are associated with large granules and marked cytoplasmic vacuolization, while type 4 correlates with large, clear keratinocytes, with small eccentric nuclei and few keratohyalin granules.

Lesions caused by HPV type 2 have a microscopic appearance similar to that of the common wart.^6^ Small intranuclear eosinophilic inclusion bodies can be observed in the cells of the upper Malpighian layer. The cells in the stratum corneum tend to retain their nuclei, which appear as round, strongly basophilic bodies surrounded by a wide clear area.^5^, ^7^

#### Verruca plana

**Figure 2:**
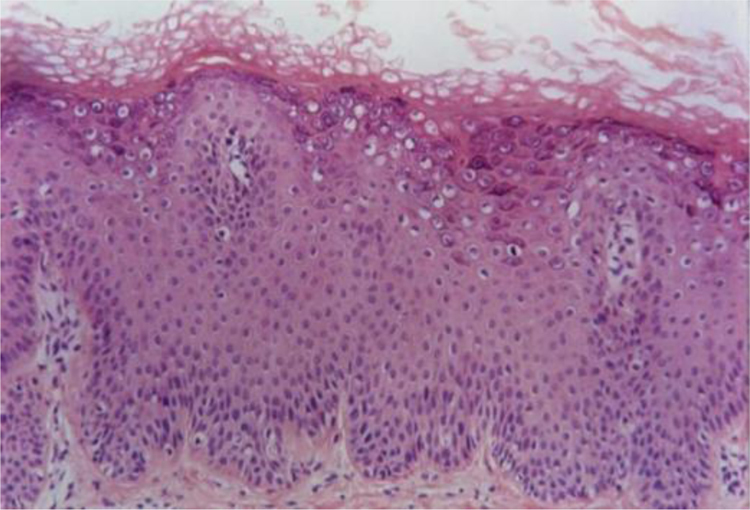
Verruca plana or flat wart, showing basket-weave hyperkeratosis, hypergranulosis, acanthosis with fusion of rete ridges and koilocytosis in the upper third of the epidermis (Hematoxylin & eosin, 100×).

Verruca plana or flat warts display hyperkeratosis and acanthosis with mild elongation of the rete ridges. A characteristic feature of these lesions is the absence of papillomatosis and parakeratosis. The cells in the upper Malpighian layer exhibit diffuse vacuolization and may be enlarged. The stratum corneum has a very evident basket-weave appearance and looks compact due to the uniform thickening of the granular layer (Fig. 2). There is no inflammatory infiltrate in the normal dermis.^5^, ^6^

#### Butcher's warts (HPV-7)

Butcher's warts, caused by HPV-7, show prominent acanthosis and small vacuolated cells with pyknosis of the centrally located nuclei. These vacuolated cells are present exclusively in the proliferated rete ridges, isolated and surrounded by strongly stained granular cells. This cytopathic effect is very characteristic of HPV-7 infection.^7^

#### Epidermodysplasia verruciformis

**Figure 3:**
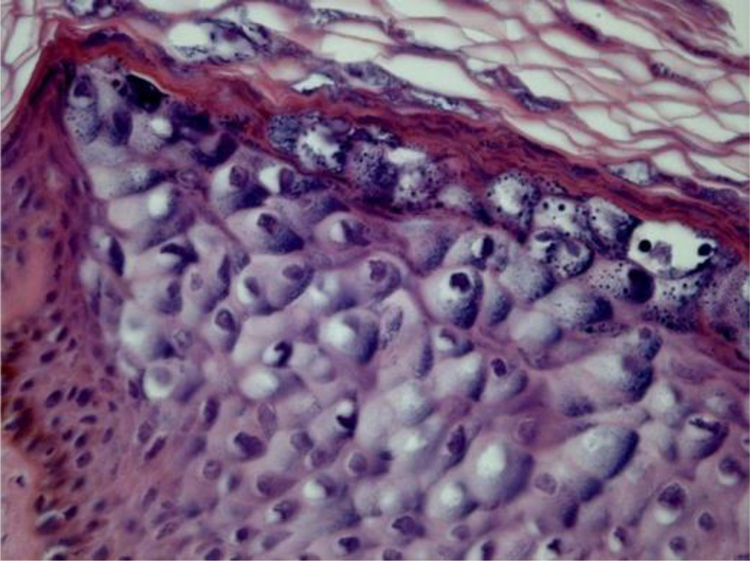
*Epidermodysplasia verruciformis* showing epithelial cells with clear cytoplasm and nuclear pyknosis (Hematoxylin & eosin, 400×).

Epidermodysplasia verruciformis (EV) lesions are characterized by stratum corneum with basket-weave appearance and keratinocyte edema. Keratinocytes have a pale appearance, are slightly basophilic and keratohyalin granules of variable size and shape, and are grouped in the granular and spinous layers of the epidermis which resembles the flat wart, but they can occupy the whole length of it. These cells have small, pyknotic nuclei, like a loose balloon in a cell vacuole (Fig. 3). It has been described that the malignant transformation in EV lesions tends to start around the hair follicles. Characteristically, the cytopathic effect disappears, and there is a predominance of dyskeratotic cells and pronounced atypia of the Bowenoid type in non-invasive and invasive areas.^5^, ^7^

### Histopathology of mucosal lesions

#### Anogenital region

Lesions located in the anogenital region include condyloma acuminatum, giant condyloma or Buschke-Lowenstein tumor, Bowenoid papulosis, Bowen's disease and Queyrat's erythroplasia. Lesion classification into low-grade squamous intraepithelial lesions (LSILs) and high-grade squamous intraepithelial lesions (HSILs) has been used to designate them according to their histopathological characteristics and clinical behavior.^6^

#### Condyloma acuminatum

Fazel et al. describe the epidermis in condyloma acuminatum (from the Greek *kondulos*/condyle and Latin *acuminatum*) as having an undulated and moderately acanthotic surface.^5^ There is a slight thickening of the corneal layer, and a prominent granular layer with coarse keratohyalin granules and koilocytes. Parakeratosis is observed in mucosal lesions.

**Figure 4:**
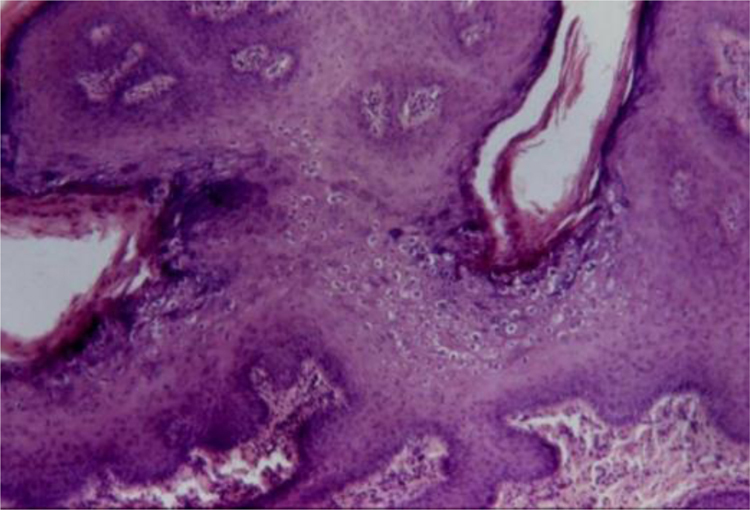
*Condyloma acuminatum* showing epithelial hyperplasia, papillomatosis, hypergranulosis and koilocytosis (Hematoxylin & eosin, 100×).

Koilocytes are keratinocytes with deeply basophilic pyknotic nuclei surrounded by a halo and clear cytoplasm, with discrete or absent keratohyalin granules. These cells tend to form aggregates on the Malpighian and granular layers, predominating in the undulation valleys (Fig. 4). There may be a similarity to seborrheic keratosis due to the occasional finding of corneal pseudocysts.

Lesions treated with podophyllin show numerous necrotic keratinocytes and a marked increase in the number of mitotic figures in metaphase in the lower half of the epidermis. Its histopathological presentation can be mistaken for squamous cell carcinoma (SCC) *in situ* or Bowenoid papulosis. The flat achromic maculae, evidenced by acetic acid, shows acanthotic epithelium and the presence of koilocytes associated with enlarged rete ridges and superficial dyskeratosis. There are, very often, varying degrees of dysplasia, with abnormal maturation, loss of polarity, increased mitotic activity and nuclear atypia. These lesions can progress to lesions such as squamous intraepithelial carcinoma or carcinoma *in situ*.^5^

The giant condyloma acuminatum of Buschke–Löwenstein exhibits mild vacuolization of keratinocytes and marked epidermal papillomatosis, with bulbous extensions of well differentiated squamous epithelium extending into the deep dermis. Keratinocytes are minimally atypical.^5^ These alterations could be observed in florid oral papillomatosis as well as in epithelioma cuniculatum.

#### Bowenoid papulosis

**Figure 5:**
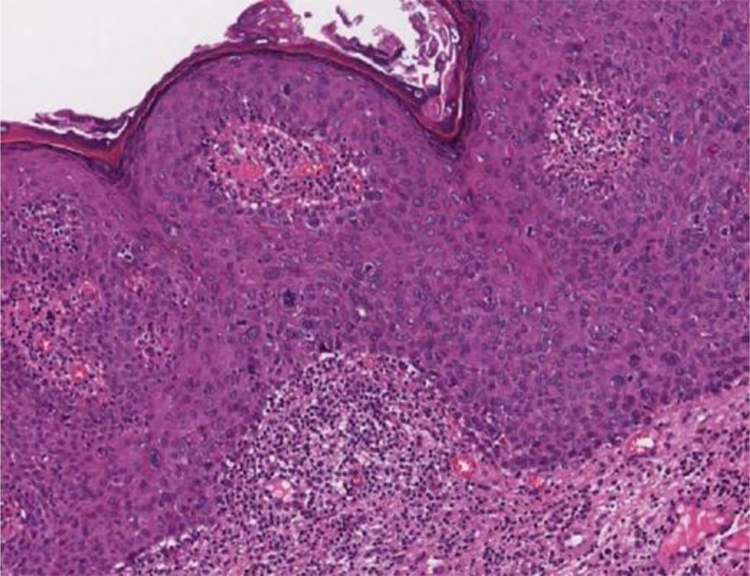
Bowenoid papulosis showing acanthosis with widening and fusion of rete ridges, loss of epidermal stratification and nuclear atypia (Hematoxylin & eosin, 100×).

In the microscopic sections, the epidermis shows psoriasiform hyperplasia and hyperkeratosis. The keratinocytes appear in clusters and show larger, hyperchromatic, pleomorphic nuclei, dyskeratosis and loss of epidermal stratification. There is an increase in the number of mitotic figures, some of them atypical, seen in all layers of the epidermis^5^ (Fig. 5). The findings are considered to be indistinguishable from SCC *in situ*, into which it can evolve.^5^, ^8^ Extracutaneous Bowen's disease and Queyrat's erythroplasia would correspond to HSILs with marked dysplasia and increased risk of progression to invasive SCC.^8^

#### Cervix

**Figure 6:**
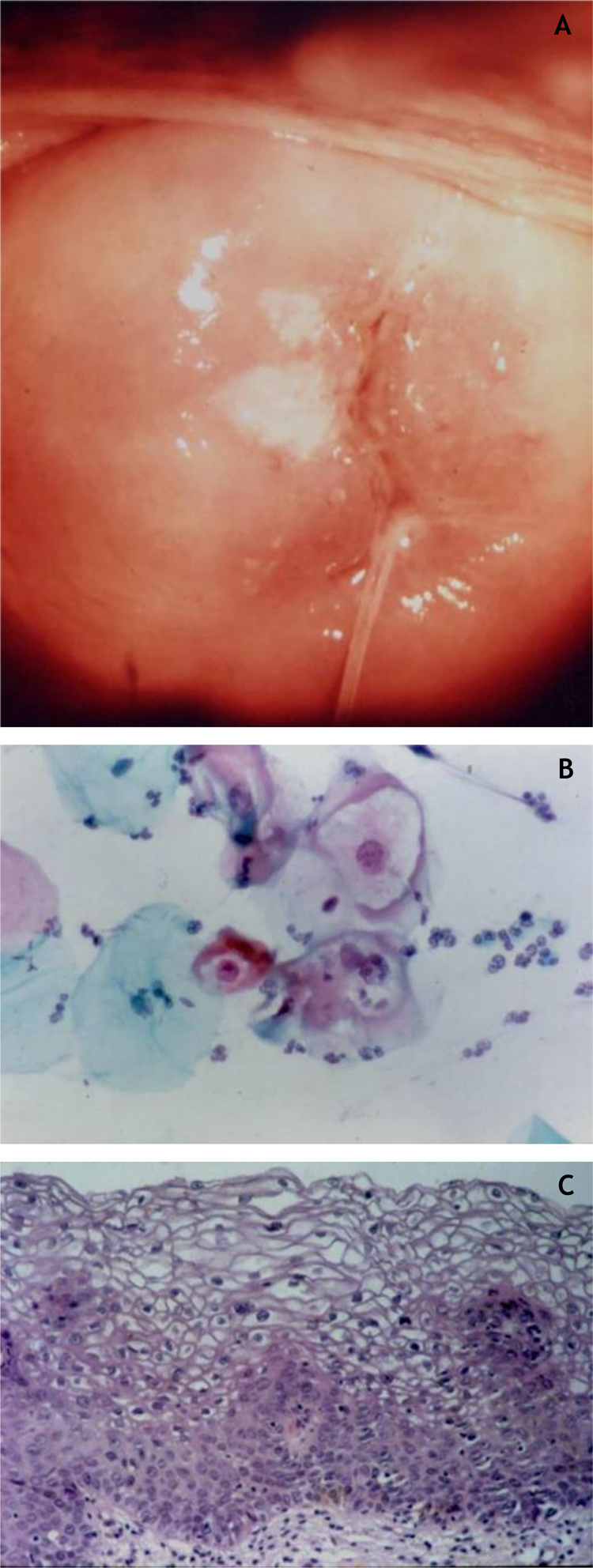
(A) Colposcopy; (B) oncotic cytopathology; (C) histopathological of the epithelium showing loss of stratification and nuclear polymorphism (Hematoxylin & eosin, 300×).

Two different types of proliferative lesions can appear in the cervix, the precursor lesions of cervical intraepithelial neoplasia (CIN) and invasive carcinoma. The knowledge of precursor lesions that show well-defined morphological characteristics allows an early diagnosis before the neoplastic invasion occurs. Clinically, they appear as acetowhite lesions (Fig. 6A) under colposcopy or with a spiculated aspect resembling condyloma.

They were called CIN and subdivided into CIN-I (mild dysplasia), CIN-II (moderate dysplasia) and CIN-III (severe dysplasia) in the same category of Carcinoma *In Situ* (CIS). Subsequently, the division of the lesions into two groups was proposed: the LSILs, which would correspond to the NIC-I, encompassing epithelial alterations suggestive of HPV infection (koilocytosis) even without dysplasia; and HSILs, comprising CIN-II and III and CIS.^9^

On histopathology, the precursor lesions of neoplasia show alterations in cell proliferation and maturation and loss of epidermal stratification, whith varying degrees of atypia. In cases of mild dysplasia, the alterations are found in the lower third of the epithelium, which shows mild cellular atypia and discrete loss of cell polarity. In moderate dysplasia, atypia and loss of polarity, in addition to cell differentiation, are more intense and comprehend half of the epithelial thickness. Mitoses are more frequent. Marked dysplasia shows alterations in the entire thickness of the epithelium with loss of stratification and the presence of typical and atypical mitoses. The finding of koilocytosis is an indicative of HPV infection (Fig. 6B and C).^10^

#### Oral mucosa

According to Betz, HPV-related benign oral cavity lesions comprise the common wart, squamous papilloma, condyloma acuminatum and multifocal epithelial hyperplasia.^11^ The histopathological characteristics of these lesions may be similar to those of other inflammatory lesions related to some syndromes, which makes communication between clinicians and pathologists important, aiming at better patient management.^11^

#### Squamous papilloma

Squamous papilloma is described as the most common benign lesion seen on the oral mucosa, both in children and adults.^11^ On histopathology, the exophytic and papillomatous architecture can be observed at low power. A central axis can be seen in some sections, or it can be suggested by the empty space between papillomatous areas covered by normal epithelium with underlying connective tissue. In the basal layer and juxtabasal area, scattered mitotic figures can be observed, but without atypias. Koilocytes can be identified in the upper spinous layer.^11^

#### Verruca vulgaris or common warts

The histopathological presentation in the mucosa is identical to that in the skin.^11^ There are exophytic projections with verrucous architecture resembling church spires and rete ridges inclined toward the center of the lesion. The granular cell layer is thickened, koilocytes have their nuclei in an eccentric location and the cytoplasm is vacuolated. These cells are frequently identified in the upper epithelial layers, in the granular layer or close to it. Mitotic figures in basal and juxtabasal areas may be present and slightly enlarged, but atypical shapes are not identified.^5^, ^11^

#### Condyloma acuminatum

In condyloma acuminatum, the lesions are papillomatous and exophytic under microscopy and, unlike squamous papilloma, the base is enlarged. The epithelium shows moderate to severe acanthosis and papillomatosis. Thick parakeratosis is often identified, with invaginations that fill up the crypts between the papillae. Similar to what has been described for anogenital condyloma acuminatum, the koilocytes are seen at the top of the spinous layer and predominate in the valleys between the papillae.^5^, ^11^

#### Focal epithelial hyperplasia (Heck's disease)

Irregular epithelial hyperplasia with mild parakeratosis is observed in focal epithelial hyperplasia. The acanthotic epithelium shows elongated, anastomosed and thickening rete ridges. Clear keratinocytes in the upper layer and binucleated cells in the medium layer are the observed histological characteristics. Ballooning degeneration can be prominent. Frequently, there is a slight lymphocytic infiltrate in the corium. Electron microscopy shows particles arranged in a crystalline pattern.^5^, ^11^

#### Electron microscopy

Electron microscopy (EM) is the technique that allows the observation of the virus in tissues. However, since the introduction of molecular biology techniques for detecting HPV DNA, these are preferred when it is necessary to prove the presence of the virus. EM is of great importance in several types of research, such as the pathogenesis of the infection, which includes considering the need for new interventions against the virus.^12^

In EM, the amount of viral particles is variable in different types of lesions, being frequently absent in common warts and in lesions that have developed into CIS or even invasive carcinoma.^13^, ^14^ The negative results do not rule out the presence of HPVs that can be detected by other methods.

Viral DNA replication occurs with basal cell proliferation. The proteins of the capsid structure appear in mid-epidermis and in the upper epidermis the mature viral structure of HPv is found. The viral particles appear as spherical bodies, with a diameter of 50 nm. Each particle consists of an electron-dense nucleoid with the aspect of an appendage surrounded by the less dense capsid.

**Figure 7:**
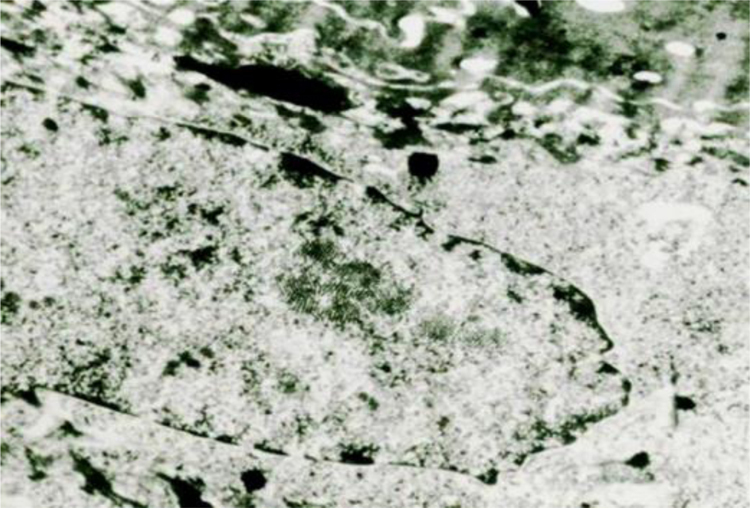
HPV electron microscopy showing intranuclear viral particles.

The viruses in the warts multiply in the nucleus, where they appear as particles in dense crystalloid aggregates (Fig. 7). In plantar warts, in the upper layer of the Malpighi stratum, their number increases, sometimes markedly, in the cells just below the stratum corneum. In flat warts caused by HPV-3, there is marked cytoplasmic edema, the tonofilaments are displaced to the cell periphery, keratohyalin granules are normal and there are numerous viral particles in the nucleus of vacuolated cells. In EV, viral particles show a semicrystalline aspect in the granular layer cell nuclei. In the Malpighi layer, edematous cells may show small virion aggregates in some cases.^14^

### Virus identification by molecular techniques

#### Laboratory procedures for HPV detection

The host's humoral immune response to HPV infection occurs in around 50–60% of individuals with infection demonstrated by a positive HPV-DNA detection test. Since it is not possible to isolate the virus using conventional culture methods, HPV identification is based on molecular methods.^15^

According to the World Health Organization (WHO), antibody screening is indicated in population-based epidemiological studies, aimed at defining pre-vaccination strategies for groups and age groups with the greatest exposure and when assessing vaccine efficacy. The use of serology as a diagnostic tool is not recommended.^15^ The analysis of the HPV type, according to the WHO and excluding specific situations, is indicated for the identification of the types associated with the appearance of neoplasms.^15^ Most trials test a pool of 12 High Risk (HR) HPV nucleic acids (16, 18, 31, 33, 35, 39, 45, 51, 52, 56, 58 and 59). Some also include HPVs 66 and 68. Some genotyping tests can differentiate HPVs 16 and 18.^16^

The first test to assess HR HPVs was approved by the Food and Drug Administration (FDA) of the United States of America in 1999, with an emphasis on the evaluation of cervical cancer. Since then, several tests have been introduced and approved and several studies involving other anogenital and oropharyngeal neoplasms have been carried out. The following tests have been approved by the FDA to evaluate cervical cancer: the Qiagen Hybrid Capture-2, approved in 2003, is a test that identifies 13 types of HR HPV: 16, 18, 31, 33, 35, 39, 45, 51, 52, 56, 58, 59 and 68; the *Cervista*, approved in 2009, identifies 14 types of HR HPVs; the *Cobas HPC*, approved in 2011, identifies HPV-16, HPV-18 and other 12 types of HR HPVs in a single reaction, and in 2014 the *Cobas 4800*, a fully automated test, was the first to be approved by the FDA as the first line screening for cervical cancer risk in women aged 25 or older; the *Aptima HPV Assay*, approved in 2011, focuses on the identification of HPV E6/E7 oncogenes through the detection of messenger RNA of 14 types of HR HPV and, finally, the *Onclarity*, approved in 2018, also detects the oncogenes E6 and E7 of 14 types of HR HPV. All of these tests show high sensitivity and specificity.^17^, ^18^ The Brazilian National Health Surveillance Agency, ANVISA, approves most of these tests, in addition to others.^19^ For an updated list, see https://consultas.anvisa.gov.br/#/saude/q/?nomeProduto=HPV.

According to the WHO, HR HPV detection tests are indicated for the screening of women aged 30 years or older, either alone or in combination with cervical cytology for the prevention of cervical cancer. These tests are more sensitive than cytology and have a higher negative predictive value. They are used for the screening of women with minimal cytological abnormalities to avoid unnecessary follow-up, to monitor women with persistent/recurrent disease and as a post-treatment test-of-cure. The WHO does not recommend testing for Low-Risk (LR) HPV.^15^

There are currently no recommendations regarding the use of screening tests for HR HPV infections in the anal, vulvar, vaginal, penile and oropharyngeal regions.^16^ HPV testing is used, with restrictions, in patients at high risk for anal cancer: screening for the p16 protein by immunohistochemistry is recommended when moderate dysplasia or precancerous lesions are found.^16^

The identification of HPV in head and neck SCCs, by *in situ* hybridization or PCR, especially in tumors of the oropharynx (tonsil, base of tongue and soft palate) can be useful, as those associated with HPV have better therapeutic response, higher survival rates and lower risk of recurrence when compared to HPV-negative patients.^16^, ^20^, ^21^

Not all women with persistent HR HPV infection or precursor lesions in the cervix, even without treatment, will progress into cancer, suggesting that the identification of HR HPV alone does not define the evolution.^15^ Therefore, other markers have been studied which are possible predictors of progression, one of them being the p16 protein. After the integration of the HR HPV genome into the infected cell, the E7 protein binds to the retinoblastoma protein (Rbp), blocking its inhibitory effect on the cell cycle. As Rbp is regulated by p16 protein, a negative feedback occurs, with increased expression of p16 protein. In the case of oropharyngeal SCCs, p16 protein screening by immunohistochemistry in tumor tissues is considered an excellent marker and an acceptable substitute for HPV screening.^21^

Moreover, regarding head and neck carcinomas, *in situ* hybridization of RNA is capable of detecting E6/E7 mRNA and allows the observation of transcriptional viral activity in tumor cells and seems to be the most specific test for HPV.^21^ Mutations in the NRF2, KEAP1 and CUL3 genes, leading to gain of function in the NRF2 pathway and in the Tp53 gene, are more prevalent among HPV-negative head and neck carcinomas when compared to HPV-positive ones. These molecular biomarkers could also be useful in patient stratification for treatment.^20^ Recent technologies are being studied aiming to detect HPV genetic material in serum and saliva, as possible non-invasive tools for the monitoring and follow-up of the treatment of patients with oropharyngeal SCC.^21^

In relation to cervical cancer, emergent biomarkers include the identification of mRNA for proteins E6/E7, screening for p16 protein, aberrant S-phase induction markers, detection of chromosomal and miRNA abnormalities; combined with advanced genotyping methods. They can be useful to differentiate those women with precancerous conditions who would need urgent treatment or diagnostic colposcopy.^22^

According to the Brazilian Guidelines for Cervical Cancer Screening, cytopathological examination is the recommended screening method for cervical cancer and its precursor lesions.^23^ Likewise, the Brazilian Ministry of Health (MH) does not recommend the routine screening for molecular evidence of HPV infection in head and neck SCC, since there is no therapeutic change based on this information.^24^

## Treatment

Speculative treatments for treating warts, especially cutaneous warts (CW) or verrucae, are widespread among the population. Successful lay experiences are suggested with absolute confidence, such as the act of blessing the region, exfoliating the lesions with coarse salt or green banana peel, acupuncture, destroying the “mother” wart so that the others will disappear, among others. The search for unusual therapies, in some situations, could be due to the esthetic appeal. The manipulation of the lesions can cause an inflammatory process and the regression of the warts, but it can also lead to rapid growth and dissemination due to trauma. The success of these approaches may be due to the spontaneous regression that is described in a significant percentage of cases, a fact that must be considered in the evaluation of any therapeutic method.^25^

Several therapeutic modalities have been used to treat HPV infections. The choice of treatment should take into account factors such as age, location, number and size of the lesions, clinical subtype and the patient's immunological status.^26^ Depending on the location, the patients’ expectations and their immunological status, an expectant approach is perfectly acceptable. However, warts that are not resolved within a year are less likely to do so spontaneously.^25^, ^27^ The ideal treatment should not leave scars, although many patients are more affected by the warts than by permanent scars.^25^

Topical treatments include predominantly destructive modalities, such as cryotherapy, chemical cauterization, surgical methods and laser removal. They can be divided into three categories: destructive, cytotoxic agents and immunotherapy.^28^ Regarding the many local treatments, it seems that scientific evidence is still scarce, even for the most recommended ones that are part of the usual therapeutic arsenal.

Otherwise, the majority of systemic treatments are based on the attempt to improve the individual's immune response to HPV. Overall, the studies are scarce and, in some situations, show controversial results. In addition, the use of these treatments ends up being made through an off-label prescription, for which the doctor must be aware and follow the necessary guidelines.

Considering this scenario, the proposal is not to exhaust the subject, but to list part of the evidence available in the literature and to offer dermatologists material that helps them in the routine care at outpatient clinics and medical offices. Table 1 shows the levels of evidence and degree of recommendation that will be discussed below (Table 1).

**Table 1:** Level of evidence and degree of recommendation of the main therapeutic modalities for skin lesions caused by HPV

	Therapeutic modality	Level of evidence^a^	Degree of recommendation^b^
Localized	Salicylic acid	1	A
	Cryotherapy	1	B
	5-Fluorouracil	2	C
	Imiquimod	3	D
	Bleomycin	2	C
	Podophyllin/podophyllotoxin	3	D
	Cantharidin	3	D
	Topical retinoid	2	C
	Trichloroacetic acid	3	C
	Cidofovir	3	D
	Surgical excision	3	D
	Lasers	2	C
	Photodynamic therapy	2	D
Systemic	Zinc sulfate	1	–
	Systemic retinoid	3	D
	H2 receptor antagonists	1	C
	Herbal treatment	2	–

Source: Created by the authors based on the references: Sterling, 2014, Goldstein, 2019 and Camargos, 2010.

a Level of evidence: 1 – Randomized Clinical Trial (RCT), Systematic Reviews (SR)/RCT meta-analysis; 2 – SR of cohort studies, cohort studies; 3 – SR of case–control studies, case-control studies; 4 – low quality case series, cohort and case-control studies; 5 – expert opinion.

b Degree of recommendation: A – experimental or observational studies with greater consistency (meta-analysis or RCT); B – observational studies with less consistency (other non-randomized clinical trials or observational or case-control studies); C – reports or case series (non-controlled studies); D – opinion without critical evaluation, based on consensus, physiological studies or animal models.

### Local treatment

#### Skin lesions

##### Salicylic acid

Formulations containing salicylic acid (SA) are considered the first line of treatment for CW by some authors.^26^ SA is a topical keratolytic agent which is easy to apply and safe, but can cause local irritation. The irritation could trigger the immune response responsible for the HPV eradication.^26^ It is superior to placebo in most studies.^26^, ^27^ There is still some controversy in the literature as to whether SA is more effective for palmoplantar lesions in relation to those located on other anatomical sites.^27^ There are several available formulations and concentrations in Brazil, reaching up to 27%, whereas the literature has descriptions of concentrations between 10% and 26%.^25^ Some formulations contain only SA, while others are associated with lactic acid.^25^ The patient can receive information and apply the medication at home, protecting the perilesional surface and repeating the process until the lesion disappears. Severe adverse events are rare.^27^

##### Cryotherapy

Cryotherapy is usually performed by applying Liquid Nitrogen (LN) on the lesion. Freezing causes tissue damage, damaging the cells and their blood supply.^27^ There is no consensus as to the freezing time or expected halo size. Any time between 10 and 30 s, with a halo of one to two mm is indicated by some authors, and these parameters may vary depending on the lesion size, location and the patient's tolerability.^25^, ^26^ More aggressive techniques seem to provide better cure rates.^27^ The applications can occur two, three or four weeks apart. The response rate at the interval of two to three weeks is higher in the first three months of treatment, but it tends to match the four-week interval when evaluating a six-month period.^25^, ^27^

Although several studies have failed to show its superiority over placebo and SA, patient satisfaction was significantly higher in the group treated with cryotherapy.^27^ Acral lesions have a better response rate when cryotherapy is associated with SA use.^27^ Ungual and periungual warts have contraindications regarding the use of this treatment modality, due to the risk of irreversible damage to the ungual matrix.^26^

##### 5-Fluorouracil

5-Fluorouracil (5-FU) has antineoplastic and antimetabolic properties that inhibit DNA and RNA synthesis, and this is believed to be the mechanism responsible for stopping aberrant epithelial proliferation.^27^ Some studies have shown superior efficacy when compared to placebo in the treatment of flat, common, palmoplantar and genital warts.^27^, ^29^ It is commonly used at a 5% concentration in a cream base, once a day, with occlusion.^25^, ^27^ The most common adverse effects include local irritation, ulcerations, allergic contact dermatitis and photosensitivity.^26^ Onycholysis is also a common side effect when treating periungual warts.^27^

##### Bleomycin

Bleomycin is an antineoplastic drug with label indications for the treatment of Hodgkin's disease and non-Hodgkin's lymphomas, malignant pleural effusion, some types of testicular carcinoma and SCCs of the oropharynx, genitals (including cervix) and skin. It is known that many SCCs, especially mucosal ones, are associated with HPV.^30^ However, the indication for the treatment of warts is not included in the package insert, so its use in Brazil would constitute an off-label use (see comments below on off-label prescription). The exact mechanism of action of bleomycin in the treatment of warts is not yet fully understood, but it seems to be associated with DNA synthesis inhibition.^26^, ^27^

Cure rates vary widely between studies (16–94%). This variation could not be associated with any drug concentration, method of application or total dose.^27^ Depigmentation and pain are the most common side effects, but edema, hyperpigmentation, erythema and scarring can also occur.^26^, ^27^ After a direct injection into the lesion, a blackened eschar may appear on the site.^26^ Some authors prefer to apply bleomycin on the wart and perform microneedling, as this method is less painful and can also be used for acral lesions.^25^, ^31^ Although there is yet no robust evidence, bleomycin can be included in the treatment of refractory lesions.^27^ It is contraindicated for pregnant and breastfeeding women.

##### Imiquimod

Imiquimod is an immunomodulatory drug that acts on the innate and adaptive response, induces the production and release of Interferon (IFN)-alpha, tumor necrosis factor-alpha and interleukin-12, in addition to promoting the activation of natural killer cells, promoting the interruption of viral replication.^25^, ^27^, ^28^ Although its use is well established for the treatment of Genital Warts (GW), there is still insufficient evidence about its effectiveness in CW.^27^ The drug is used at a 5% concentration and varying doses have been proposed in studies.^27^, ^28^ Erythema, edema and erosions may occur.^27^ It can be used in combination with destructive methods in an attempt to reduce local recurrence.^28^

##### Topical retinoids

Tretinoin is the main retinoid used in the treatment of CW, usually at a dose of 0.05% in a cream base. Its use is generally reserved for cases with multiple recalcitrant lesions or patients with flat warts.^25^

##### Podophyllin/podophyllotoxin

Podophyllin is an antimitotic agent that can cause tissue necrosis. It must be applied by the physician, at concentrations of 10–25%. Systemic effects may occur if it is applied on large body surfaces. It is contraindicated during pregnancy. Although it is used in GWs, it is not yet well established in CWs. Podophyllotoxin can be administered by the patient and causes less irritation than podophyllin.^25^ Although it appears in the list of drugs available for HPV treatments in the public health network, podophyllotoxin is not found in Brazil.^1^

##### Cantharidin

Cantharidin is a vesicant agent that causes acantholysis. Due to its mechanism of action, it carries a reduced risk of scarring. Its application is practically painless, and the discomfort appears only 24 h later. It is highly toxic if administered systemically, so it must be applied in the medical office. The most often used concentration is 0.7%.^25^ Vakharia et al. showed high efficacy of a formulation containing cantharidin, salicylic acid and podophyllotoxin for the treatment of plantar warts in adults and children. However, further studies are required to confirm this result.^32^

##### Cidofovir

Cidofovir is a nucleotide analog that works by inhibiting viral DNA polymerase. The treatment is carried out with monthly intralesional injections. There are studies underway to evaluate the use of a cream-based formulation. Only local side effects have been reported: pain, burning, pruritus and erythema.^28^

##### Immunotherapy

Topical immunostimulatory agents have been studied for decades in an attempt to elucidate their therapeutic role in the treatment of recalcitrant warts. Dinitrochlorobenzene has been used in the past with promising results, but it is no longer indicated due to its mutagenic potential.^26^, ^28^ Diphencyprone has been used for this purpose because it is more potent and less mutagenic. The application of 0.001% diphencyprone to glabrous skin and 0.0001% to other regions showed a good response in previously sensitized patients.^28^

Several other substances have been described as possible immunotherapy agents that can be applied in the treatment of CWs: PPD, *Candida* sp. antigen, triple viral vaccine (MMR), topical zinc sulfate, among others. However, further studies are needed to determine their role in the management of cases.^27^, ^28^

##### Destructive methods

Surgical excision is a method that removes CWs using a curette, scalpel or electrocautery.^33^ It is widely used in the treatment of CWs, although many dermatologists contraindicate it, due to its morbidity and high recurrence rates.^27^ Filiform warts on the face seem to respond better to this treatment modality.^26^

The use of photodynamic therapy with aminolevulinic acid to treat plantar warts has been described, but studies have shown contradictory results regarding the effectiveness of this intervention.^27^ The CO_2_ laser is described as a destructive method with potential for use in CWs, as well as the pulsed dye laser; however, more studies are needed to determine the effectiveness of these methods.^27^

### Anogenital lesions

#### Genital warts

The treatment of GWs can be carried out in a single session, using destructive methods such as electrocoagulation, surgical excision or CO_2_ laser.^1^, ^34^ These techniques are associated with a high probability of total lesion resolution, but with higher recurrence rates than the other therapies.^35^

Trichloroacetic Acid (TAA) (80–90%), podophyllin (10–25%) and cryotherapy are options for treatments that require multiple applications at the medical office.^1^ Podophyllotoxin 0.5% seems to be the most effective and relatively low-cost agent, but it is contraindicated during pregnancy.^1^, ^34^ TAA is a caustic agent that promotes destruction by chemical coagulation. It must be applied only to the lesions.^1^ It is associated with fewer side effects than cryotherapy on this location.^35^ Imiquimod, podophyllottoxin and 5-FU are viable options for home treatment.^1^

#### Premalignant and malignant lesions

##### Bowenoid papulosis

In the therapeutic approach, it should be kept in mind that although histopathology shows many atypias, the evolution of these lesions is favorable in most cases. Spontaneous regression frequently occurs, especially in young and male individuals. It can be more aggressive in the elderly and immunocompromised individuals.^36^, ^37^ Treatment modalities include surgical excision, electrocoagulation, cryotherapy with LN, CO_2_ laser and, in selected cases, Mohs micrographic surgery. Imiquimod also seems to be a viable option. The use of radiotherapy for malignant and pre-malignant lesions associated with HPV is not recommended.^38^

##### Verrucous carcinomas

Minimally invasive surgery, together with local therapy, is adequate for patients with small lesions or high intraoperative risk. Likewise, the association of cryotherapy with topical chemotherapy can be considered. Mohs micrographic surgery can also be indicated. The main treatment remains an extensive surgery, with broad resection and often reinterventions to complete the excision. Complementing the surgical treatment for small lesions can be carried out with laser application and local or intralesional treatments with 5-FU and bleomycin, associated with cisplatin and methotrexate; TAA, cidofovir and imiquimod are other described options. In addition to these, systemic immunotherapy with IFN-alpha and systemic chemotherapy with several drug combination regimens have been used in selected cases. The use of radiotherapy is controversial.^33^, ^39^

### Special situations

#### Pregnancy

TAA is the treatment of choice for pregnant women with localized lesions, and cryotherapy with LN is also indicated. For patients with extensive affected areas, surgical treatment is the preferred conduct.^1^ Podophyllin, podophyllotoxin and imiquimod are contraindicated during any stage of pregnancy.^1^

#### Immunosuppressed patients

The management of immunosuppressed patients is a challenging situation. Treatment may not result in the complete healing of the lesions, but it helps to reduce their size and the esthetic impact. Therapeutic options are the same as those previously reported; however, variation and association of methods may be necessary.^25^

#### Systemic treatment

##### Cimetidine

Cimetidine, an H2 receptor antagonist antihistamine, has been used in several clinical studies for CW, with conflicting results, some showing significant improvement and others showing disappointing results.^40^ The use of this medication has also been reported for the treatment of HPV skin lesions in patients with EV, with little improvement.^41^ Some side effects described are: headache, dizziness, diarrhea, skin rash, urticaria, alopecia, gynecomastia, mastalgia, arthralgia and myalgia.^40^

##### Levamisole

Levamisole, which has several immunomodulating effects, has been used in a few controlled studies for the treatment of CWs with conflicting results. Its side effects include: nausea, dysgeusia, skin rash, alopecia and the flu-like syndrome.^40^

##### Trace elements

Zinc, an important trace element in the body immune function, was compared to a placebo, at a dose of 10 mg/kg/day, in a few studies for the treatment of resistant CWs: some with good results, showing an improvement of up to 87% and others with similar results between the drug and the placebo.^40^

A prospective, double-blind, randomized Brazilian study compared cimetidine 35 mg/kg/day (maximum 1200 mg/day) and zinc sulfate 10 mg/kg/day (maximum 600 mg/day), during three months for the treatment of CWs in 18 patients. Zinc sulfate was more effective than cimetidine, with complete response in 62.5% of patients and partially in 25%. The study limitation is the small sample size.^42^ The most common side effects were nausea, vomiting and epigastric pain.^40^

Selenium has been studied as prophylaxis for the appearance of HPV-related lesions in Solid Organ Transplant (SOT) recipients but it has not been shown to be effective.^41^

##### Echinacea

Echinacea, a perennial herbaceous plant that belongs to the *Asteraceae* family, has three species with therapeutic properties: *E. purpurea*, *E. angustifolia* and *E. pallida*. It is used for the prevention and treatment of upper airway infections, and it has also been tested for the treatment of CWs in a few studies, with conflicting results.^40^

##### Interferon

IFN, a cytokine produced by T lymphocytes, fibroblasts and other cells, has immunomodulatory, antiproliferative and antiviral properties and can be used to treat HPV lesions using topical, intralesional or systemic formulations. A systematic review carried out in 2009 included 12 controlled and randomized studies that comprised 1445 patients with genital warts treated with INF. When compared to the placebo, the topical use of IFN showed a statistical difference (RR = 2.68; 95% CI 1.79–4.02 and *p* < 0.000001), but the systemic use did not (RR = 1.25; 95% CI 0.80–1.95 and *p* > 0.05).^43^ There have been reports of IFN use in the treatment of patients with warts and immunosuppression, such as EV (pegylated IFN alpha-2b and recombinant IFN alpha-2a), idiopathic CD4+ T cell lymphocytopenia (IFN gamma-1b and INF alpha-2b) and HIV-positive patients (pegylated IFN alpha-2b).^41^ The use of IFN-alpha in the treatment of recurrent respiratory papillomatosis, as an adjuvant therapy, is controversial.^44^ The most common side effect of systemic use is a flu-like syndrome with fever, chills, headache, myalgia and fatigue.^40^

##### Retinoids

Acitretin, a retinoid for systemic use, has had its utilization reported (alone or in combination with IFN) in the treatment of HPV-related skin lesions of patients with EV. The response is variable, being good in some cases, but showing recurrence after treatment interruption. As for etretinate, reports have shown a minimal or transient response.^41^ There has been a report on improvement in GWs in a patient with systemic lupus erythematosus using systemic isotretinoin associated with surgical treatment.^41^

##### Cidofovir

Cidofovir, an acyclic nucleoside phosphonate that acts on viral DNA polymerase, which has been approved for systemic use in cytomegalovirus (CMV) retinitis in HIV-positive patients, has had reports of topical and intralesional use in the treatment of anogenital lesions caused by HPV in transplant recipients; moreover, there have been reports of improvement of refractory skin lesions in HIV-positive patients with its topical use, albeit with high rates of recurrence.^41^

In recurrent respiratory papillomatosis, it has been used through several application routes: intralesional, systemic and inhaled. Although widely used, the treatment results are variable and refer to its combined use with surgical treatment. Systemic use is limited by the effects of renal toxicity and risk of oncogenicity. Other adverse reactions described are: skin rash, headache and vocal cord scarring.^44^

##### Other considerations

The use of bevacizumab, a monoclonal antibody that targets vascular endothelial growth factor (VEGF) receptors, has had several reports on the improvement of recurrent respiratory papillomatosis, with both intralesional and systemic use. Controlled, randomized clinical trials and studies with larger case series must be carried out to assess its efficacy and safety.^44^

A relevant aspect to be considered concerns the immunosuppressant used in transplant recipients: calcineurin inhibitors, such as cyclosporine, pimecrolimus and tacrolimus, are associated with the development of warts, especially if associated with azathioprine, while mTOR inhibitors, such as sirolimus, temsirolimus and everolimus, are related to the improvement and reduction of warts and associated malignancies. There are reports of kidney and liver transplant recipients with warts that showed improvement when these immunosuppressive medications were changed.^41^

New drugs targeting molecular pathways have been studied in the treatment of HPV-related diseases: molecular inhibitors directed at the DNA binding activities of HPV proteins E1 and E2 or at the anti-apoptotic activities of oncogenes E6 and E7; proteasome and histone deacetylase inhibitors in addition to E6 and E7 therapeutic protein vaccines.^45^

The off-label prescription of medications is characterized by the use of a drug, by the physician, when its use has not been approved by the health agencies in situations that are not included in the package insert, for instance, regarding its dosage, interval between doses, age group, diseases and even the stages of the same disease. Indications for using a certain medication vary from country to country and throughout the time of drug use, new indications may be authorized by agencies based on scientific evidence.

In Brazil, ANVISA draws attention to the fact that this type of prescription is carried out at the risk and discretion of the physician who prescribes it, which may eventually characterize a medical error. However, it also points out that, in most cases, it is an essentially correct use, which has just not been approved yet.

Regarding the treatment of HPV manifestations, many of the treatments will be carried out through an off-label prescription. Physicians should be aware of this possibility and should always share this information with their patients and, preferably, ask them to sign the free and informed consent form for the medication use.^46^

## Prevention

### Vaccines

The relationship between HR HPV and cervical cancer (CC) has been well established, and this is one of the most common neoplasms in women from low- and middle-income countries.^30^, ^47^ This context makes the prophylactic vaccine against HPV to be considered as one of the most important advances in the field of women's health.^48^

It is considered that almost all cases of CC are caused by HPV infection, which also occurs in 90% of anal neoplasms. Moreover, HPV is related to neoplasms of the vagina, penis, vulva and oropharynx, with varying percentages, and is the cause of GWs and recurrent respiratory papillomatosis.^30^, ^47^

All three prophylactic vaccines currently available against HPVs are obtained by recombinant DNA technology from the viral L1 protein, which will constitute the virus-like particles, or VLP.^47^ The available vaccines are bivalent, quadrivalent and nonavalent, and all of them contain types 16 and 18 (HR HPV), with the quadrivalent including LR HPV 6 and 11, and the nonavalent including, in addition to the aforementioned ones, types 31, 33, 45, 52, and 58.

In Brazil, the quadrivalent vaccine was introduced in the SUS (Unified Health System) network for girls between 9 and 14 years of age in 2014. In 2017, the use was extended to boys, and it is currently recommended for the age range between 11 and 14 years old in this group.^1^, ^49^ In this age range, regardless of gender, two doses are recommended with a six-month interval between them. In Brazil, the vaccine is also recommended for people living with HIV/AIDS, solid-organ and bone marrow transplant recipients and cancer patients aged 9–26 years old, under medical prescription. In the latter group, three doses of the vaccine are recommended with a two-month interval between the first and the second dose and six months between the first and the third dose (0, 2 and 6 months).^1^

The three vaccines are considered safe and among the associated adverse effects are episodes of syncope, attributed not to the components of the vaccine itself, but to the vaccination process. Especially among the younger population, syncope has been described with the application of other vaccines, blood collection and parenteral administration of medication.^49^ Local reactions have also been described, especially with the nonavalent type.^30^ The severe adverse effects reported, such as neurological, autoimmune diseases and thromboembolism, have not had a causal association attributed to the HPV vaccines.^47^, ^49^

There is robust evidence of individual and population benefit in reducing both benign lesions and lesions caused by HR HPVs after HPV vaccination.^1^, ^30^ Antibody formation after vaccination is higher among individuals who have not started sexual activity and, therefore, were not exposed to HPVs, as well as among younger individuals. The immunogenicity duration is not known; however, protection persistence for up to ten years with the quadrivalent vaccine and, for at least six years, with the nonavalent vaccine has been reported.^30^, ^47^

A major challenge is to maintain vaccination coverage at levels considered to be adequate, even for the first dose. In Brazil, there was a reduction of almost 23% in the coverage of the target population (girls) between the years 2014 and 2015 for the first dose of the vaccine (92% × 69.5%). In 2017 the data showed percentages below 80% for girls at the first dose (79.2%) and only 48.7% at the second dose. For boys in that same year, the coverage at the first dose was of 43.8%.^48^ As it is not known whether a single dose of the vaccine would provide sufficient protection, it could be considered that low vaccination coverage may compromise in the future not only individual but collective health, regarding the prevention or not of malignant or pre-malignant HPV related diseases.^47^, ^48^ Low vaccination coverage among boys is a matter of concern, as even considering that heterosexual men can benefit from the herd immunity resulting from the vaccination of women, the same does not apply to Males who have Sex with Males (MSM). Therefore, vaccination coverage at appropriate levels is necessary, regardless of gender.^47^

The vaccination of individuals older than 26 is not recommended for all individuals. However, some adults between the ages of 27 and 45 may decide to take the HPV vaccine based on discussion with their physician, if they have not been adequately vaccinated when they were younger. In this age group, vaccination offers fewer benefits, for the abovementioned reasons. The vaccination prevents new HPV infections but does not treat existing infections or diseases.^50^, ^51^

The vaccine is contraindicated during pregnancy. If pregnancy occurs after the first dose, or if it is inadvertently administered during pregnancy, the subsequent dose should be suspended and the vaccination schedule should be completed after delivery. Breastfeeding does not contraindicate vaccination.^50^ Other contraindications would be: for the bivalent vaccine, a history of allergy to latex, because when previously packaged in syringes, these may have components with latex residue; for the quadri- and nonavalent vaccines, a history of allergy to *Saccharomyces cerevisiae* yeasts present in its composition. People with acute illnesses with moderate to severe conditions should have their vaccination postponed.^51^

The difficulties in implementing HPV vaccination are many, which have different and sometimes dynamic characteristics, with specific situations that require prompt intervention so that myths and misinformation are not disseminated.^48^ Some of these aspects are discussed in the Health Education topic.

### Health education

In health education aimed at HPV prevention, it is necessary to consider not only its relevance due to the prevalence/incidence in the populations, but also its transcendence. The burden of individual suffering associated to morbidity and mortality should be highlighted, which leads to feelings of fear and the experience of stigma for many individuals. Moreover, it translates into high costs for public health.^52^

When considering health education focused on skin manifestations, one should bear in mind that in immunocompromised patients, skin cancer develops at an earlier age, is more aggressive and carries a greater risk of metastasis, which implies the need to adopt effective preventive measures for this population.^25^ Although it is the task of all health professionals, the importance of the dermatologist in the prevention, identification and treatment of pre-neoplastic lesions and neoplasms in this population is undeniable. Similarly, the propaedeutic, diagnostic, therapeutic and preventive approach to most of the clinical manifestations of HPV in the mucosal membranes and the viral infection itself is also the responsibility of dermatologists, along with other health professionals.

Knowledge about the sexual behavior of the population in which one intends to develop health education activities is essential. Regarding sexuality, a population survey carried out in Brazil that included 12,000 people aged between 15 and 64 years old aiming to assess the knowledge, attitudes and practices related to STIs and viral hepatitis, showed that a quarter of them reported having started sexual activity before the age of 15, 34.9% of men and 15.4% of women.^53^ Among young individuals up to 24 years old, 35% started before 15 years old. 43.9% reported having had more than 10 sexual partners throughout their lives, especially among men (56.6% × 26.3%). Approximately 10% of men and 6% of women reported some STI symptom during their lifetime. Only 71% of women had undergone oncotic cytopathology in the past three years. Among those who had sex in the past 12 months, almost 28% had more than one partner and 12% had more than five partners. Condom use is low; among young people aged 15–24, 61% used condoms at their last sexual intercourse, but only 39% did so when considering all respondents.^53^

In relation to HPV, health education should cover the dissemination of knowledge about infection, related diseases and their possible consequences, more specifically, the oncogenic potential of the virus. Considering the existence and availability of the quadrivalent vaccine in SUS and the evidence of its efficacy in reducing genital warts and pre-malignant lesions related to HPV, it is essential to ensure, during this process, the acceptance and access to the specific vaccination.^1^, ^48^, ^54^ In the broadest sense, health education aimed at preventing HPV and other STIs aims to improve the tools of individuals with a view to guaranteeing their autonomy in relation to their own sexual health and that of their partners.^1^

The fundamental points highlighted in studies on the subject are the knowledge, attitudes and practices related to sexuality, especially among adolescents and young adults, who constitute the most vulnerable group for STIs/HPV, as well as for early pregnancy.^52^, ^55^, ^56^ Although it is considered that variables such as age group, gender, having received the HPV vaccine and even the type of instrument used in the research may influence the results, it is a consensus that knowledge is the basis to establish safer attitudes and practices^54^, ^55^, ^57^

Knowledge about HPV is insufficient in several studies, both in Brazil and in other countries.^52^, ^54^, ^55^, ^57^ For instance, not considering that condom use can prevent STIs and pregnancy and not knowing about the HPV vaccine were reported by almost half of those investigated in two studies in Brazil.^52^, ^55^ Regarding the attitudes, the belief that it is not necessary to use condoms in stable relationships and even feeling offended when its use is suggested by the partner is consistent with the practice of not using them by a significant percentage of young individuals interviewed in Brazil.^52^, ^53^, ^55^

As with several other diseases, lower schooling is related to less knowledge, which translates into greater vulnerability according to Fontes et al.^52^ On the other hand, these same authors, when defining a scale of knowledge, attitudes and practices, found some relevant social determinants aiming at reducing vulnerability, among them the interest in learning, talking with parents and teachers about their sexuality and having some of these figures as a reference, not abusing alcohol, having leisure activities and participating in social work, as well as having an interest in learning and making conscious use of the internet.^52^

The adoption of a safe behavior in sexual relationships implies previous knowledge about what constitutes risk. Knowing the importance of using condoms, being able to negotiate their use with sexual partners, or even having the option to postpone or abstain from sexual involvement are behaviors, attitudes and practices that are learned and necessary for the prevention of STIs and pregnancy.^56^, ^58^

The interventions to disseminate/consolidate knowledge about STIs and lead to safer attitudes and practices are relevant, and in recent years they have gained an ally with great potential for reaching these individuals, through digital technologies.^58^, ^59^ These are considered more accessible and acceptable and their effects are comparable to those in person.^58^, ^59^ In a meta-analysis that included 16 studies and 11,525 young individuals, there were no differences in the assessed outcomes regarding the attitudes and practices between technology interventions and control programs.^58^ The duration of the technology interventions remains a question for discussion, but their cost/effectiveness is a strong argument.^58^, ^59^ The challenge that remains for the interventions is to prolong their effects on attitudes and practices over time.^56^, ^58^

Another meta-analysis that evaluated 63 studies and included almost 60,000 individuals in several countries showed that almost 75% of the interventions were based on health promotion theories.^56^ In the short term, there was a significant impact on knowledge related to sexual health, attitudes and practices, knowledge that remained with a significant decrease in the medium term. In the long term, interventions only improved condom use.^56^ This study showed that interventions aimed at preventing STIs and teenage pregnancy increased the use of condoms when performed in countries with a higher HDI (Human Development Index), in a school environment, with a theoretical basis and by not promoting sexual abstinence.^56^ This is a problem that becomes very relevant and requires health professionals’ commitment regarding the current conservative public policies, particularly in Brazil.

The stigma related to STIs can hinder the search for treatment and protection methods, in addition to increasing the individual's burden of suffering. It is admitted that individuals with little knowledge feel more stigmatized when they are diagnosed with HPV infection.^60^ A qualitative study, based on information obtained from a website (Experience Project), showed that the negative perception of one's image was the type of stigma prevalent among people.^60^ This fact could hinder the search for knowledge from appropriate sources about the infection, leading people, in most situations, to deal with the focus on the problem and not on the emotions, with doubts limited to symptoms and the side effects of the disease treatment.^60^ The authors recommend using the virtual environment to conduct forums and debates in order to demystify the misconceptions about HPV.^60^

Regarding the vaccine, the knowledge about HPV goes beyond the target population itself, since most of them should be vaccinated in childhood or early adolescence.^1^ The barriers to implementing adequate vaccine coverage are considered to be multifactorial. These include a low level of knowledge about HPV and diseases associated with it, the resources offered by the vaccine, unfounded concerns of parents and policy makers, cultural barriers and costs to the health care system.^48^ Additional difficulties would be the lack of routine preventive consultations for adolescents, the absence of immediate benefits for the child/adolescent, and not requiring proof of vaccination for school enrollment.^61^

The lack of information by parents and guardians about the importance of vaccination in the pre-pubertal stage coupled with the belief that the vaccine could lead to early sexual initiation or promiscuous behaviors can lead to the postponement of the vaccine or even non-vaccination. However, these arguments are known to have no scientific basis.^50^, ^61^ Despite all the evaluations and experience accumulated with vaccines over the years, their safety is frequently questioned, their side effects are feared and sometimes, fancifully associated to vaccine.^50^, ^51^

The anti-vaccine movement should not be underestimated. Coping strategies on several fronts need to be permanently worked on, considering the multi-causality of these barriers. The importance of the role schools and health professionals have for vaccination success is recognized and needs to be encouraged. The physician must use all medical care opportunities to check and/or indicate HPV vaccination.^47^, ^57^, ^61^

## Financial support

None declared.

## Authors’ contributions

Marcelo Grossi Araújo: Study conception and design; literature search and critical reading of the articles; writing of the manuscript; critical review of the manuscript and final approval of the submitted version.

Geraldo Magela Magalhães: Study conception and design; literature search and critical reading of the articles; writing of the manuscript;, critical review of the manuscript and final approval of the submitted version.

Lucas Campos Garcia: Study conception and design; literature search and critical reading of the articles; writing of the manuscript; critical review of the manuscript and final approval of the submitted version.

Érica Cristina Vieira: Study conception and design; literature search and critical reading of the articles; writing of the manuscript; critical review of the manuscript and final approval of the submitted version.

Maria de Lourdes Ribeiro de Carvalho Leite: Study conception and design; literature search and critical reading of the articles; writing of the manuscript; critical review of the manuscript and final approval of the submitted version.

Antônio Carlos Martins Guedes: Study conception and design; literature search and critical reading of the articles; writing of the manuscript; critical review of the manuscript and final approval of the submitted version.

## Conflicts of interest

None declared.
